# Commentary: Help-Seeking Patterns and Attitudes to Treatment amongst Men Who Attempted Suicide

**DOI:** 10.3389/fpubh.2016.00088

**Published:** 2016-05-10

**Authors:** Leo Sher

**Affiliations:** ^1^James J. Peters Veterans’ Administration Medical Center, Bronx, NY, USA; ^2^Icahn School of Medicine at Mount Sinai, New York, NY, USA

**Keywords:** men’s mental health, help-seeking, suicide

The *Journal of Mental Health* has published an article, “Help-seeking patterns and attitudes to treatment amongst men who attempted suicide” by Cleary (1). The author followed up 52 men, aged between 18 and 30 years, who made a medically serious suicide attempt for 6–8.5 years. The author reported that at the time of discharge from a hospital all patients were referred to psychiatric outpatient services but one-third (32.7%) never presented and 20% attended only for a short period of time. Almost half (48%) of the sample made a subsequent attempt, and 12% completed suicide.

Suicide in men is a difficult issue and has been described as a “silent epidemic” (2–5). In the United States and Canada, men die from suicide attempts three to four times more often than females (6). Male suicide rates are larger than female rates at all ages (6). From 1999 to 2010, the suicide rate among Americans aged 35–64 years grew by almost 30%, to 17.6 deaths per 100,000 people, up from 13.7% (7). While suicide rates are increasing among both middle-aged men and women, much more men take their own lives. In 2010, the suicide rate for middle-aged men was 27.3 deaths per 100,000, whereas for women, it was 8.1 deaths per 100,000. In Europe, men commit suicides 3–10 times more frequently than women (4, 6). The gender-suicide gap is especially large in some Eastern European countries, such as Poland, Hungary, Lithuania, Latvia, Ukraine, and Belarus. After accidents, suicide is now the second most common cause of death in 15- to 24-year-old males in the United Kingdom, and it is even the leading cause of death in several other European countries in this age group (8).

Lack of help seeking among men is one of the most important issues in the field of men’s mental health (9). It contributes to high suicide rates in men. Cleary (1) states that “Factors contributing to low take-up of services include lack of awareness of psychiatric symptoms, reluctance to disclose distress and negative attitudes to seeking professional help.” All three mentioned factors indicate that mental and non-mental health professionals should do a better job evaluating, educating, and treating men. This is the most important conclusion that can be drawn from the study by Cleary (1) who pointed out that “A considerable level of unmet need is implied here and young males may need more assertive outreach” and “More nuanced training for frontline, especially A&E/‘Accident and Emergency’/, staff in suicidal behaviour would both improve attitudes and increase detection rates for those with serious suicidal intent.” Indeed, men’s lack of help seeking may be related to a lack of training, interest, and responsiveness from some psychiatrists, other mental health and non-mental health clinicians who sometimes treat depressed, anxious, and/or suicidal men in an incompetent and unempathic manner (4). Clinicians often do not diagnose mood disorders and/or suicidality in men, because they have a history of impulsive–aggressive behavior and/or alcohol and drug abuse that misleads to diagnoses of personality disorders and/or substance use disorder.

Among men, and young men, especially, masculinity is directly associated with receiving less health care and particularly psychiatric services (3, 4, 9–11). Public health education interventions targeting misconceptions among males that a lifestyle involving poor self-care and lack of help seeking is masculine are needed. Men should be encouraged to use the available resources such as community mental health clinics, self-help, and peer-support groups. In the United States, military veterans can use the facilities of the Veterans’ Health Administration system (12). In summary, better education of both medical professionals and the general public about men’s mental health issues is necessary and may reduce suicidality in men.

## Author Contributions

The author confirms being the sole contributor of this work and approved it for publication.

## Conflict of Interest Statement

The author declares that the research was conducted in the absence of any commercial or financial relationships that could be construed as a potential conflict of interest.
